# Long admission waiting list at the Orchard clinic-why?

**DOI:** 10.1192/bjo.2021.763

**Published:** 2021-06-18

**Authors:** Hannah Sayeed, Johanna Brown, Fionnbar Lenihan

**Affiliations:** 1 ST6 Forensic Psychiatry, the Orchard clinic, NHS Lothian; 2 Clinical director and Consultant Forensic psychiatrist, the Orchard Clinic, NHS Lothian; 3 Consultant psychiatrist, St Bricin's Military Hospital

## Abstract

**Aims:**

The Orchard clinic is one of the three medium secure units in Scotland. This project was completed:

- To gain an understanding of the causes of the Orchard clinic's long admission waiting list.
- To use this information to improve current clinical pathways, service development and further research and development.

**Method:**

To study the longitudinal traffic flow through the clinic from January 2017 to December 2019, data were collected for this time retrospectively from electronic minutes of fortnightly bed management meetings at the Orchard clinic.

This was cross checked with the Orchard clinic's record of admissions and discharges during this time and approved by the Forensic Research and Audit Group, NHS Lothian.

**Result:**

November 2018 onwards, a surge of 90% was observed in the admission waiting list.

Looking at the trends of traffic flow through the clinic during this time, the following observations were made:

1. More admissions than discharges, especially November 2018 onwards.

2. New referrals for medium secure care at the Orchard clinic peaked twice during this time.

3. Delayed discharges peaked in July 2018 and further in January 2019 running parallel to the surge in admission waiting list thereafter.

4. 42% patients on the delayed discharge list belonged to other health boards awaiting local low secure/community placements.

**Conclusion:**

Delayed discharges were identified as a constant parallel to the long waiting list and hence identified as the main factor contributing to it. Out of area (non-NHS Lothian) admissions were noted to be linked to these delayed discharges.

Regular peaks in new referrals was also noted to be contributory.

